# Burn and thoracic trauma alters fracture healing, systemic inflammation, and leukocyte kinetics in a rat model of polytrauma

**DOI:** 10.1186/s13018-019-1082-4

**Published:** 2019-02-19

**Authors:** Lauren H. Mangum, Joshua J. Avila, Brady J. Hurtgen, Alicia L. Lofgren, Joseph C. Wenke

**Affiliations:** Extremity Trauma and Regenerative Medicine, US Army Institute of Surgical Research, San Antonio Military Medical Center, JBSA Ft Sam Houston, San Antonio, TX USA

**Keywords:** Polytrauma, Extremity fracture, Inflammation, Nonunion

## Abstract

**Background:**

Singular traumatic insults, such as bone fracture, typically initiate an appropriate immune response necessary to restore the host to pre-insult homeostasis with limited damage to self. However, multiple concurrent insults, such as a combination of fracture, blunt force trauma, and burns (polytrauma), are clinically perceived to result in abnormal immune response leading to inadequate healing and resolution. To investigate this phenomenon, we created a model rat model of polytrauma.

**Methods:**

To investigate relationship between polytrauma and delayed healing, we created a novel model of polytrauma in a rat which encompassed a 3-mm osteotomy, blunt chest trauma, and full-thickness scald burn. Healing outcomes were determined at 5 weeks where the degree of bone formation at the osteotomy site of polytrauma animals was compared to osteotomy only animals (OST).

**Results:**

We observed significant differences in the bone volume fraction between polytrauma and OST animals indicating that polytrauma negatively effects wound healing. Polytrauma animals also displayed a significant decrease in their ability to return to pre-injury weight compared to osteotomy animals. Polytrauma animals also exhibited significantly altered gene expression in osteogenic pathways as well as the innate and adaptive immune response. Perturbed inflammation was observed in the polytrauma group compared to the osteotomy group as evidenced by significantly altered white blood cell (WBC) profiles and significantly elevated plasma high-mobility group box 1 protein (HMGB1) at 6 and 24 h post-trauma. Conversely, polytrauma animals exhibited significantly lower concentrations of plasma TNF-alpha (TNF-α) and interleukin 6 (IL-6) at 72 h post-injury compared to OST.

**Conclusions:**

Following polytrauma with burn injury, the local and systemic immune response is divergent from the immune response following a less severe singular injury (osteotomy). This altered immune response that follows was associated with a reduced capacity for wound healing.

**Electronic supplementary material:**

The online version of this article (10.1186/s13018-019-1082-4) contains supplementary material, which is available to authorized users.

## Background

Fracture healing is a complex process that requires the early involvement of the immune system to generate a local inflammatory process necessary to drive the initiation of regeneration. This initial inflammatory response is followed by a resolution phase and quickly progresses toward the proliferation and remodeling phase [1]. Delayed or ineffective bone fracture healing represents a significant clinical problem, often requiring multiple readmissions and surgical revision, as well incurring higher cost of care and loss of productivity [2, 3]. Certain patient populations often experience delayed or non-union following fracture, particularly patients with impaired or restricted immune function due to co-morbidities, such as those with diabetes mellitus and osteoporosis, as well as smokers or the elderly [4, 5]. Severe trauma or polytrauma patients represent an additional subset of immunologically impaired individuals, presenting with a pronounced systemic inflammatory response that is associated with decreased fracture healing [1, 6]. Polytraumatic injuries often present with injury severity scores of > 15 and consist of a combination of burn injuries, bone fractures, blunt force trauma, hemorrhage, ischemia/reperfusion, surgery, or infection. Under normal healing conditions, only 5–10% of fractures fail to heal, while the incidence of delayed and non-union is significantly higher when a patient suffers from multiple injuries [6, 7]. In such severe injuries, the immune response itself can lead to detrimental host tissue damage and death and is suspected to adversely affect fracture healing [8].

Upon fracture, local damaged blood vessels rupture, leading to the formation of a hematoma, while the surrounding injured tissue releases cytokines and chemokines to recruit neutrophils, macrophages, and lymphocytes to the site of injury. The recruited immune cells induce the initial inflammatory processes required for repair, and loss of an appropriate, sequential immune response has been demonstrated to contribute to disturbed bone regeneration [9–12]. Following the formation of the hematoma, polymorphonuclear neutrophils (PMNs) are rapidly recruited to the site of injury to clear apoptotic cells and debris [12]. These cells are short lived, surviving only a few hours to 1 day and are followed by the recruitment of resident and circulating macrophages [12]. Though neutrophils have been described as necessary for the initiation of healing [9], persistence of PMNs within the wound space and failure to recruit macrophages to the wound site has been implicated in the delay in bone fracture healing [1, 8, 13]. A recent study demonstrated significant changes to the concentrations of inflammatory cytokines and mRNA associated with the immune response within the fracture hematoma and surrounding bone marrow of immunologically restricted (IR) patients compared with normal patients, and the authors hypothesized that these local changes to the immune response could be responsible for delayed healing outcomes in this patient population [4]. The systemic inflammation induced by additive trauma has been demonstrated to elicit similar changes to those seen in IR patients. In a rat model of osteotomy and thoracic trauma, increased infiltration and persistence of PMNs as well as increased expression of IL-6 was noted within the periosteum of the fracture callus, while infiltration of macrophages was significantly delayed and reduced [8].

Injury is the most common cause of death and disability of young adults and children and represents a small, care-intensive proportion of trauma patients. A recent review of European trauma registries found that approximately 10–16% of all trauma patients present with polytraumatic injuries. For individuals with significant injuries to more than one region of the body, traffic-related accidents were the main mechanism of injury, accounting for 65–82% of all injuries across age groups [14]. Additionally, increased utilization of high-energy explosive devices in recent armed conflicts have caused a dramatic increase in the rate of polytraumatic injuries compared to previous conflicts [15, 16], with the majority of these cases involving injury to multiple organs or regions of the body with soft tissue and extremity damage, bone fracture, vascular damage, or burn wounds [17, 18].

Previous studies have demonstrated that additive concurrent trauma can delay fracture healing, but few animal models have included a large total body surface area burn as an additive trauma and the impact of thermal injury on fracture healing has not been well characterized [19, 20]. While the cellular and immune responses to fracture have been investigated under conditions of normal healing or in immunologically restricted patients, the transcriptional patterns associated with the immune response and osteogenesis present within the fracture site in a model of polytrauma have yet to be described. To investigate these specific parameters and establish a rat model of additive trauma, we have developed a polytrauma model which provides substantial levels of insult through a combination of a 3-mm non-critical sized femoral osteotomy, which is a controllable and reproducible surrogate for fractures, blunt force trauma to the chest, and a full thickness scald burn. While other models of multiple injury and fracture healing have utilized blunt chest trauma [8, 21, 22] or traumatic brain injury (TBI) [23], the addition of a thermal injury to our model introduces an additional source of perturbation to the immune system [24] and attempts to recapitulate an injury pattern that has not been addressed in the literature. Furthermore, the contribution of an early increase in damage-associated molecular patterns (DAMPs), specifically HMGB1, to the onset of immunosuppression in multi-trauma patients has only recently been described [25]. The purpose of this study is to characterize the early local and systemic immune response, as well as alterations to expression of osteogenic markers, in a setting of severe polytrauma to compare to previously published data. This work is intended to establish a useful model of battlefield injuries, or severe traumatic injuries in a civilian population, for the purposes of evaluating interventional strategies to improve wound healing outcomes.

## Methods

### Animals

All animal procedures were approved by the Institutional Animal Care and Use Committee at the United States Army Institute of Surgical Research and were conducted in compliance with the Animal Welfare Act and in accordance with the principles of the Guide for the Care and Use of Laboratory Animals. Male outbred Sprague-Dawley rats weighing 385 ± 2 g were grouped housed preoperatively in a pathogen-free vivarium at the United States Army Institute of Surgical Research which is accredited by the Association for Assessment and Accreditation of Laboratory Animal Care. All animals had food and water provided ad libitum and unrestricted activity prior to and after all procedures. Animals were divided into two cohorts (*n* ~  8/group) of trauma representing an increased ISS as follows:

A. Osteotomy (OST)
B. Osteotomy, blunt chest trauma, and full-thickness burn (polytrauma).

In some experiments (leukocyte populations), OST and polytrauma groups were also compared to naïve animals (*n* = 8) to analyze departure from baseline.

### Surgery

Pre-surgical provisions included the use of pre-procedural analgesia and induction agents. Sustained release buprenorphine (Buprenorphine-HCL SR Lab 1.2 mg/kg, SC) was given at least 15 min prior to trauma to maintain adequate analgesia for 48–72 h after the procedure [26]. Anesthesia was induced and maintained with 1–3% isoflurane and oxygen delivered via a nose cone on a Bain circuit connected to the rodent gas anesthesia machine (VetEquip Inc., Pleasanton, Ca). Animals were monitored to ensure a surgical plane of anesthesia was maintained at all times. All surgical sites were shaved and prepared with alcohol and betadine. Naïve animals did not undergo any experimental manipulation and were utilized to serve as a source of undamaged tissue for comparison to tissue collected from trauma groups.

### Postoperative care

Animals did not receive any prophylactic antibiotics. Following surgery, each animal was assessed for signs of pain or distress twice a day for the first 72 h and weekly thereafter, evaluating general appearance, behavior, body weight, clinical signs of wound infection (including the osteotomy site and scalded area), and gait. No animals displayed signs of clinical infection at any point during this study. If the animals were found to be in distress, additional Buprenorphine-SR was administered and additional assessments were performed until resolved. If animals were found to have more than 10% body weight loss relative to surgery, 3 mL of normal saline was given subcutaneously once a day and additional enrichment was provided until resolved. Due to concerns regarding the use of chronic analgesia and bone fracture healing [27], analgesics were administered at signs of breakthrough pain throughout the course of the study (Additional file 1).

### Osteotomy

**Fig. 1:**
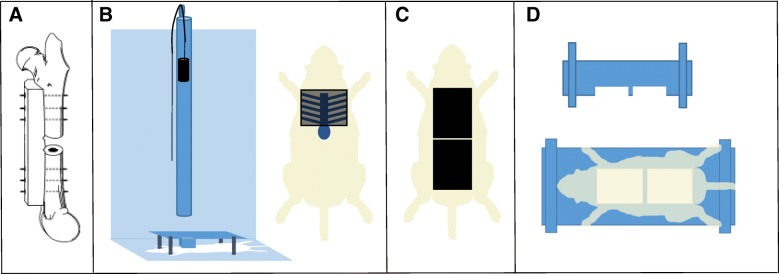
Representative images of surgical and injury methods. **a** Osteotomy was achieved via internal fixation with a polyacetal plate and threaded K-wires. **b** Blunt chest trauma device and placement of animal in apparatus to ensure all energy is directed toward the lungs. **c** Scalded area on the dorsal side of the animal. **d** Scald burn plexiglass mold

A 3-mm non-critical sized osteotomy was performed as described previously described using aseptic techniques, with minor modifications (Fig. 1a) [28–30]. This defect size has been found to be non-critical in rats [31] and will heal without intervention in normal healthy animals after approximately 8 weeks. The right femur was exposed through a 3-cm lateral incision, and the musculature was bluntly separated from the bone. The periosteum was stripped away from the bone and a pre-drilled polyacetyl plate (27 × 4 × 4 mm) was placed on the anterolateral surface of the femur and fastened proximally and distally with six 0.9-mm-diameter threaded Kirschner wire (K-wire) inserted orthogonally to the long axis of the bone. Following stabilization, an osteotomy was created in the midshaft of the femur using a reciprocating saw under continuous irrigation of normal saline to prevent thermal damage in a manner similar to procedures previously described by our labs [32, 33]. The soft tissue was then closed with absorbable sutures and skin clips. Directly after closure, confirmation of plate and pin placement combined with the osteotomy was visualized by radiograph using a cabinet X-ray system (Ultrafocus 100, Faxitron Bioptics, LLC). The animals in the osteotomy only group were immediately recovered in their cages and survived for either 4 h, 24 h, 72 h, 10 days, or 5 weeks. At the designated time point, animals were anesthetized and euthanized with an overdose of FatalPlus®, and the limb harvested and processed for RT^2^ PCR or μCT and histology.

### Blunt chest trauma

**Fig. 2:**
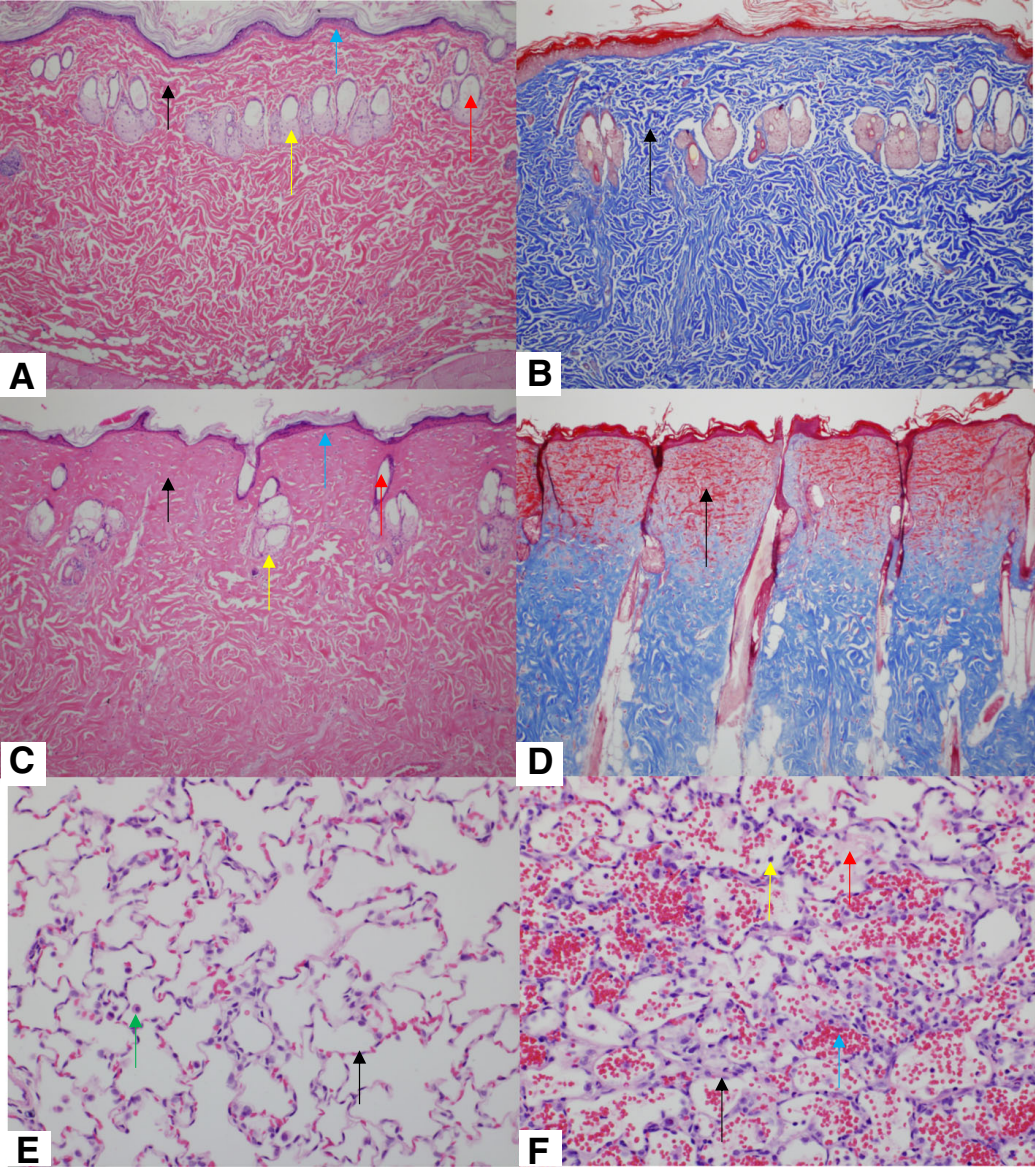
Injured and non-injured lungs and skin. Representative images (H&E and Masson’s Trichrome) from **a** normal skin (naïve) (H&E; 100x). **b** Normal skin (naïve) (Masson’s Trichrome; × 100). **c** Scalded skin (H&E; × 100). Note the coagulated stroma (black arrow), necrotic epidermis (blue arrow), necrotic hair follicle (red arrow), and necrotic sebaceous gland (yellow arrow) compared to panel **a**. **d** Scalded skin (Masson’s Trichrome; × 100). Note that the coagulated stroma stains red compared to the normal stroma (black arrow) in panel **b**. **e** Normal lung (naïve) (H&E; × 400). **f** Lung from a rat subjected to blunt force trauma (H&E; × 400). Note the thickening of the alveolar septa with fibrin and inflammatory cells (black arrow) and alveoli which contain hemorrhage (blue arrow) and fibrin (red arrow) and mixed with inflammatory cells (yellow arrow). Note that alveoli normally contain few macrophages (panel **e**; green arrow), but are not associated with alveolar septal lesions, hemorrhage, or fibrin

While still in a surgical plane of anesthesia, blunt chest trauma was generated by the energy transfer from a falling weight to a platform resting on the animal’s chest, which generates standardized bilateral lung contusions (Fig. 1b) [34]. Briefly, a 0.3-kg weight was dropped from a known height (67.6 cm) to exert ~ 2.0 J of energy on the animal’s chest while in dorsal recumbancy, assuming all potential energy is transferred from the weight to the animal and excludes any friction of the device. Normal respiration was confirmed prior to moving forward to the next trauma. Histology of the lungs following blunt chest trauma was visualized 24 h post-trauma using H&E staining (Fig. 2e, f).

### Full-thickness burn

Full-thickness burns were performed utilizing a protocol developed at the United States Army Institute for Surgical Research as previously described, with minor modifications (Fig. 1c, d) [35]. After blunt chest trauma, rats were maintained in a surgical plane of anesthesia and prepared for a full-thickness burn injury. The dorsal side of each rat was shaved to remove fur and expose the dermis layer. Rats were then placed in a specialized Plexiglas mold to expose approximately 20% of the total body surface area and partially submerged in 100 °C water for 10 s. Immediately following the scald procedure, the exposed surface was blotted dry with sterile absorbent material. The animals were immediately recovered in their cages with continued monitoring and survived for either 24 h, 72 h, 10 days, or 5 weeks. At the designated time points, animals were anesthetized and euthanized with an overdose of FatalPlus®, and the limb harvested and processed for RT^2^ PCR or microcomputed tomography (μCT) and histology.

To confirm full-thickness burn, histology of the burn wound area was performed at 72 h post-trauma. Biopsies of normal (Fig. 2a, b) and burned skin (Fig. 2c, d) were formalin fixed, paraffin embedded, sections were cut at a thickness of 8 μm, deparaffinized, and stained with hematoxylin and eosin (H&E) and Mason’s trichrome and visualized by light microscopy.

### Microcomputed tomography and histology of the femoral defect

**Fig. 3:**
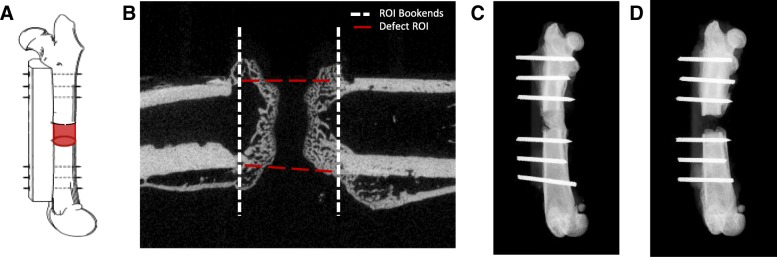
μCT and radiographic images of OST and polytrauma. **a** Representation of μCT volume of interest. **b** ROI bookends encompassed the central 251 slices in between the first distal and proximal slice that did not include cortical bone. **c** Representative radiograph of the 5-week osteotomy group (*n* = 8). **d** Representative radiograph of the 5-week polytrauma group (*n* = 7)

Microcomputed tomography (μCT) scans (VivaCT40, Scanco Medical) were performed on a set of harvested rat femurs at 5 weeks post-injury (*n* = 8 and 7 rats for OST and polytrauma groups, respectively). Osteotomy regions were scanned at high resolution with a 10.5-μm voxel size at a voltage of 70 kVp, a current of 114 μA, and an integration time of 300 ms. The images were converted to 8-bit bitmap files using Image J (National Institutes of Health, Bethesda, MA) and reoriented with DataViewer (Bruker-MicroCT, Kontich, Belgium) so that the slices were in the plane of the defect border. Regions of interest (ROI, Fig. 3a, b) and three dimensional analysis were completed using CTAn (Bruker-MicroCT, Kontich, Belgium).

A volume of interest of 251 slices was analyzed (~ 2.6 mm) centered within the osteotomy defect, and a polygonal area was drawn around the intact cortex proximal and distal to the defect and interpolated throughout the slices to simulate the area in which the intact femur should lie (Fig. 3a, b). A global threshold was calculated using the Otsu method [36], and a value of 93 was determined to be the delineation between non-mineralized and mineralized tissue. Three dimensional image reconstructions were used to quantify bone and values are reported as bone volume fraction (BV/TV) (%), which is the ratio between bone volume and tissue volume within a given volume of interest, was calculated for both treatment groups as previously described [30].

Histological analysis was conducted on femurs at 24 h and 72 h post-trauma (*n* = 6/group). Femurs were immediately harvested following euthanasia and stored in 10% neutral buffered formalin for approximately 7 days for fixation. Femurs were rinsed in type 1 ultra-pure water and stored in formic acid bone decalcifier (Immunocal, Decal Chemical Corp, Tallman NY) for approximately 7 days. Femurs were embedded in paraffin and cut in longitudinal section at a thickness of 8 μm, deparaffinized, and stained with H&E. Histologic analysis of the osteotomy (fracture site), K-wire insertions (pin sites), and bone marrow adjacent to the fracture site on each sample was performed using light microscopy. The pin and fracture sites were evaluated for the severity of inflammation, i.e., infiltration of neutrophils and macrophages, and the number of osteoclasts and osteoblasts on a scale of 0 to 4 (i.e., 0 = normal; 1 = minimal; 2 = mild; 3 = moderate, and 4 = severe). The presence of granulation tissue, determined by increased mitotic rate of fibroblasts, was also analyzed at the pin insertion sites. Bone marrow necrosis at the fracture site was scored on a scale from 0 to 4 (i.e., 0 = none; 1 = 10%; 2 = 11–25%; 3 = 26–40%; 4 = > 40%). All slides were reviewed and scored by a board-certified veterinary pathologist. Prior to analysis, images were converted to black and white at 8-bit and analyzed on open access software (Image J, NIH).

### RNA isolation and RT^2^ PCR array

Immediately after animals were euthanized, the osteotomy site was excised, placed in RNA*later®* stabilization reagent (Qiagen, Valencia, CA), and stored according to manufacturer’s directions. Disruption and homogenization was performed with 750 μl of QIAzol lysis reagent. Samples were incubated at RT for 5 min. Following incubation, 150 μl of chloroform was added, samples were mixed, incubated for 3 min, and the aqueous phase separated by centrifugation. Aqueous phase was transferred to a sample tube and RNA extraction was performed utilizing silica-coated magnetic-particle pre-filled reagent cartridges (EZ1 RNA Tissue Mini Kit, Qiagen, Valencia, CA) including 10-μl RNase-free DNase I and loaded into the EZ1 Advanced XL (Qiagen, Valencia, CA) using the pre-programmed EZ1 RNA Universal Tissue protocol. Samples were eluted into 50 μl of RNase-free water. The concentration of RNA was determined at OD_260/280_ using a spectrophotometer (NanoDrop 8000, Thermo Scientific, Wilmington, DE), and ribosomal RNA band integrity was analyzed utilizing a RNA ScreenTape on a 2200 TapeStation (Agilent, Santa Clara, CA). Samples with RNA Integrity Numbers of approximately 7 were selected for analysis. First strand complementary DNA (cDNA) was synthesized from RNA using 172 μg of total RNA and the RT^2^ First Strand Kit (Qiagen, Valencia, CA) according to manufacturer’s directions. Pathway-focused gene expression analysis was performed for innate and adaptive immune responses (catalog number PARN-052Z, Qiagen, Valencia, CA) and osteogenesis (catalog number PARN-026Z, Qiagen, Valencia, CA) from the local fracture site and performed according to the manufacturer’s instructions. Gene expression was performed on a real-time PCR detection system (CFX96, Bio Rad, Hercules, CA) and data was analyzed using web-based software (www.SABiosciences.com/pcrarraydataanalysis.php). Comparisons were made between OST and polytruama at 24 and 72 h for the innate and adaptive immune response (*n* = 5 /group) and at 10 days post-trauma for osteogenesis (*n* = 6/group).

### Blood collection

Whole blood was collected at terminal time points of 24 h (*n* = 8/group) and 72 h (*n* = 6/group) post-trauma from a deeply anesthetized rat by cardiac puncture, followed by euthanasia. Complete blood cell counts were analyzed on a hematology analyzer (COULTER A^c^•T diff2 Hematology analyzer, Beckman Coulter, Brea, Ca), for circulating concentration and composition of lymphocytes, monocytes, and granulocytes (*n* = 8/group 24 h post-injury; *n* = 7, 72 h post osteotomy; *n* = 6, 72 h post polytrauma; *n* = 8 naïve). Additional aliquots of blood were collected in EDTA tubes; plasma was isolated and stored at − 80 °C for analysis.

### DAMPS and cytokines

Circulating HMGB1 at 6 h (*n* = 5/polytrauma and *n* = 3/OST) and 24 h (*n* = 5/polytrauma and *n* = 8/OST) post-trauma was assessed via a high sensitivity in vitro enzyme-linked immunosorbent assay (ELISA; HMGB1, 6010, Chondrex, Redmond, WA) according to manufacturer’s directions. In addition to HMGB1, 23 plasma cytokines were quantified at 24 h and 72 h using a commercially available multiplex kit (Rat Cytokine Group 1 Panel 23 Plex, Bio-Rad Laboratories, Inc., Hercules, CA). Samples were run in triplicate, according to manufacture directions (*n* = 5 rats/group/time point).

### Statistical analysis

Statistical analysis was conducted using GraphPad Prism 7.01 (GraphPad Software Inc., La Jolla, CA). Deficit of bone regeneration within the segmental defect of polytrauma animals compared to OST was assessed by one-tailed Student’s *t* test. Differences between WBC groups were assessed by one-way ANOVA with Tukey’s Post-hoc analysis at a *p* < 0.05 level of significance. Differences between cytokines were assessed by one-way ANOVA with Sidak's multiple comparisons test  at a *p* < 0.05 level of significance. Differences between groups regarding histology scoring were statistically tested using a Mann-Whitney *U* test at a *p* < 0.05 level of significance. Fold changes to gene transcription were calculated and generated using the RT2 PCR array data analysis web portal (https://dataanalysis.qiagen.com/pcr/arrayanalysis.php). Genes with differences greater than 2 fold (*p* < 0.05) compared to control group were considered significant.

## Results

### Delayed bone fracture healing in polytrauma injury

The extent of bone healing was assessed by microcomputed tomography by comparing bone volume fraction between the OST and polytrauma cohorts 5-week post-trauma. Polytrauma was hypothesized to be detrimental to bone healing, and therefore, fracture healing in this injury group was compared to standard osteotomy by one-tailed Student’s *t* test. Animals receiving polytrauma injury exhibited a significant decrease in the bone volume fraction throughout the defect area (6.73% ± 1.53) compared to OST (13.48% ± 2.94), as determined by one-tailed Student’s *t* test.

### Cellular accumulation in fracture site and surrounding area

**Fig. 4:**
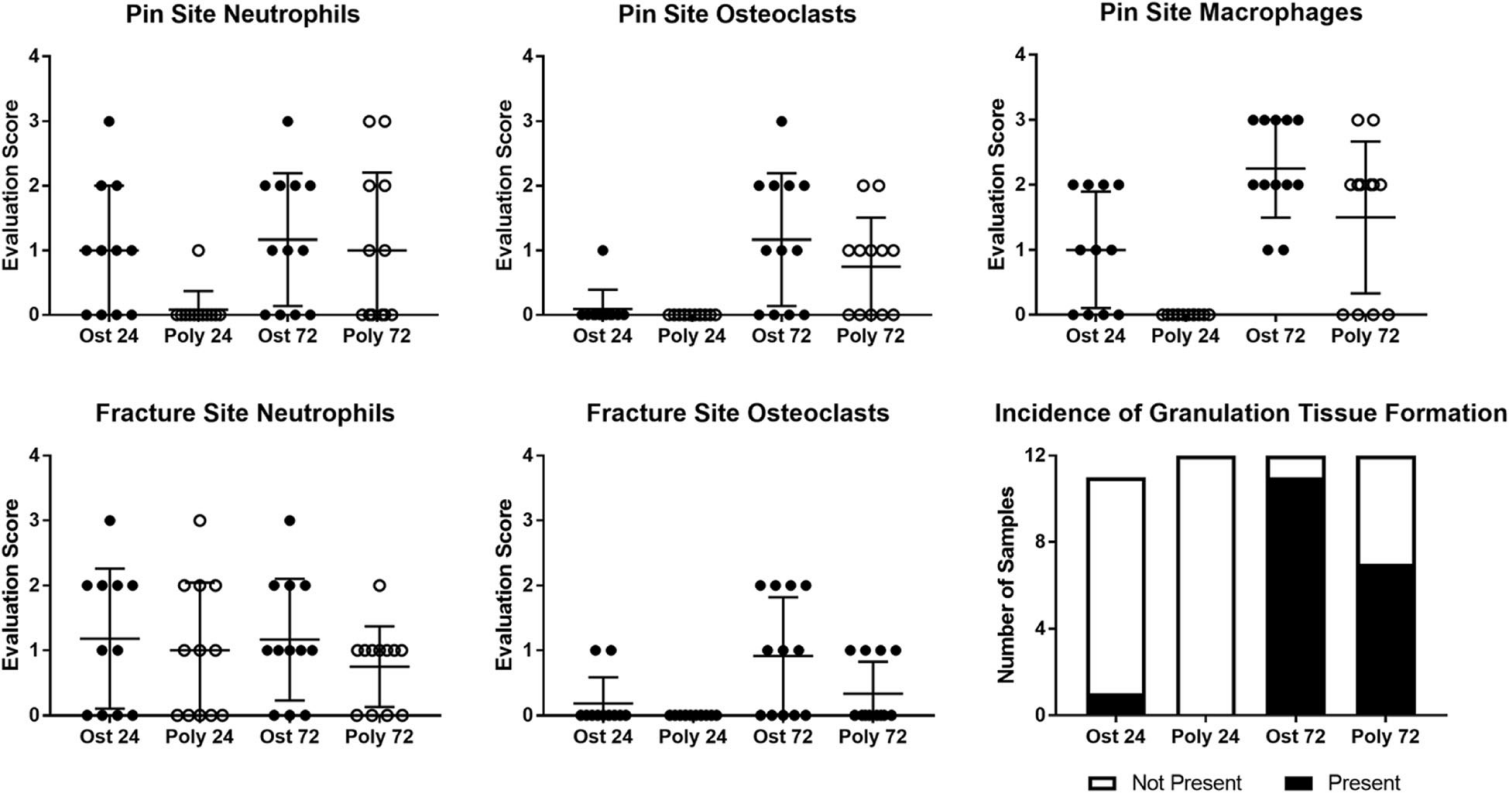
Quantification of immune cell infiltration into pin site and fracture site. Histological analysis was conducted on femurs at 24 and 72 h post-trauma (*n* = 6/group). Femur sections were cut at a thickness of 8 μm, deparaffinized, and stained with hematoxylin and eosin (H&E) for analysis. The degree of immune cell infiltration and inflammation scored by a veterinary pathologist on a scale of 0–4 and compared between the osteotomy and polytrauma groups within each time point. Significances were determined by Mann-Whitney test, *p* < 0.05; *n* = 11–12 animals per time point

Previous studies of polytrauma and isolated fracture found significant differences in the number of macrophages, PMNs, and osteoclasts within the periosteum at the fracture callus [8]. In this study, the osteotomy gap, pin sites, and bone marrow were visualized by light microscopy and the degree of immune cell infiltration and inflammation scored by a veterinary pathologist on scale of 0–4. Comparisons were made between the OST and polytrauma groups at specified time points. Marrow necrosis was not significantly different between the injury groups at either time point, as determined by the Mann-Whitney test. There were also no differences detected in the formation of granulation tissue or the number of osteoclasts at the pin site at 24 or 72 h post-trauma. However, at 24 h, the number of macrophages and neutrophils around the pin was significantly reduced in the polytrauma group. The number of macrophages quantified at the pin site remained lower at the 72-h time point, but this was not significant (*p* = 0.061) (Mann-Whitney test, *p* < 0.05) (Fig. 4).

**Fig. 5:**
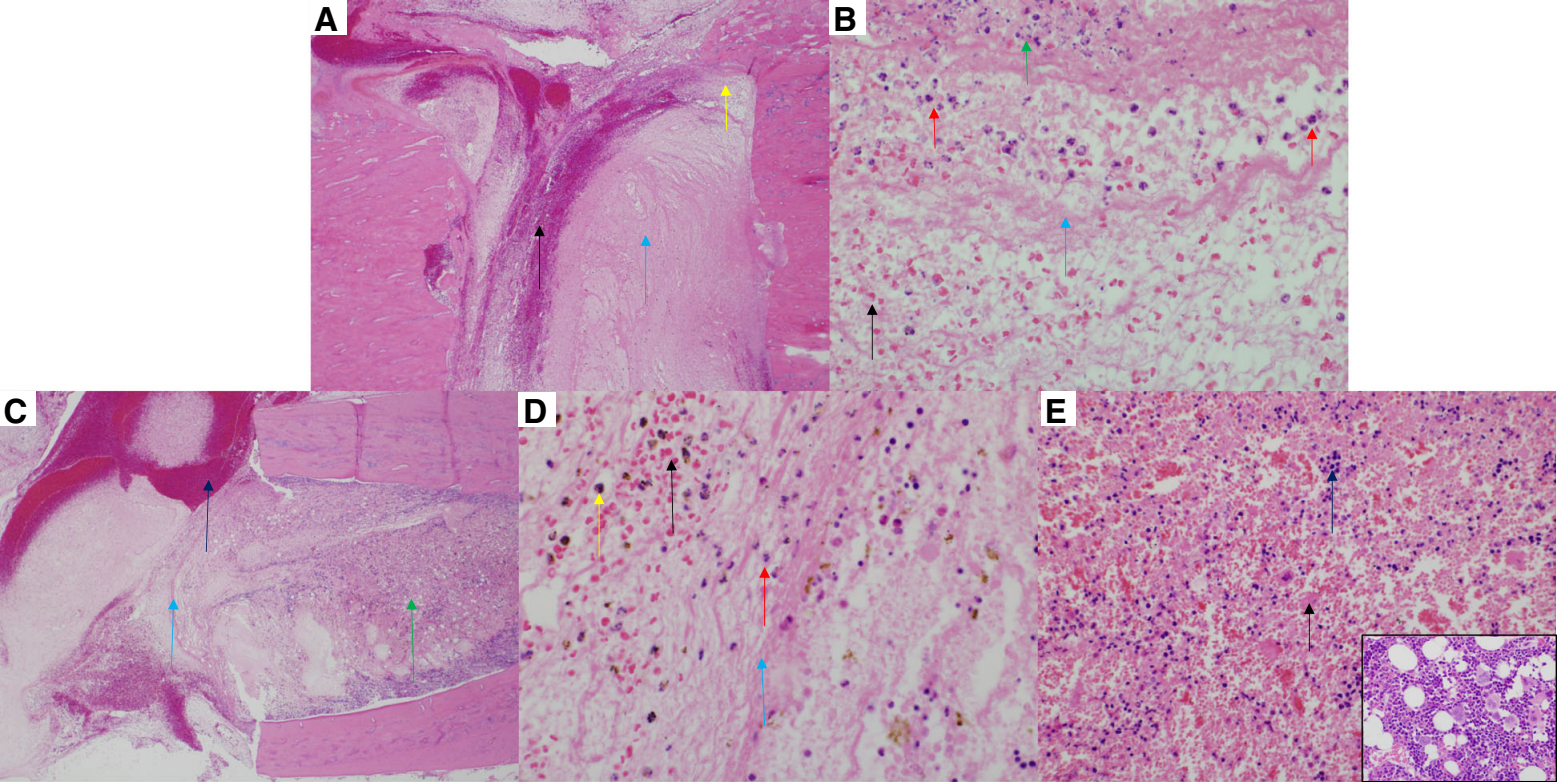
Osteotomy site in a rat femur (H&E) at 72 h post-trauma. Sections of the osteotomy site (*n* = 6/group) were stained with hematoxylin and eosin stain. **a** Representative images of the fracture site from the osteotomy group. There is no callus formation. The osteotomy site is filled with moderate hemorrhage (black arrow) and marked fibrin (blue arrow) (× 40). **b** Higher magnification (× 600) of the site identified by the yellow arrow in (**a**). Note the degenerate neutrophils (red arrows), karyorrhectic and cellular debris (green arrow), fibrin (blue arrow), and hemorrhage (black arrow). **c** Representative image of the fracture site from the polytrauma group. There is no callus formation. The polytrauma osteotomy site is filled with marked hemorrhage (black arrow) and fibrin (blue arrow) (× 40). **d** Higher magnification (× 600) of the site identified by the blue arrow in **c**. Note the accumulation of fibrin (blue arrow), hemorrhage (black arrow), few neutrophils (red arrow), and hemosiderin-laden macrophages (orange arrow). The presence of hemosiderin-laden macrophages is indicative of phagocytosis of erythrocytes and hemoglobin. **e** Higher magnification (× 400) of the bone marrow identified with the green arrow in **c**. Note the cellular necrosis (black arrows) and marked loss of the cells and adipose tissue compared to the inset (normal bone marrow from sham; × 600)

Within the fracture gap, there were no significant differences in the number of neutrophils between the groups at 24 or 72 h, while macrophages could not be quantified at this site. In polytrauma animals, no osteoclasts or osteoblasts were present within the fracture site at 24 h. Animals which underwent an osteotomy only exhibited minimally increased osteoclasts and osteoblasts within the fracture gap at 24 h compared to polytrauma; however, there were significantly more osteoclasts within the fracture gap of the osteotomy-only animals by 72 h (Mann-Whitney test, *p* < 0.05) (Fig. 5) [8].

### Circulating WBC levels

**Fig. 6:**
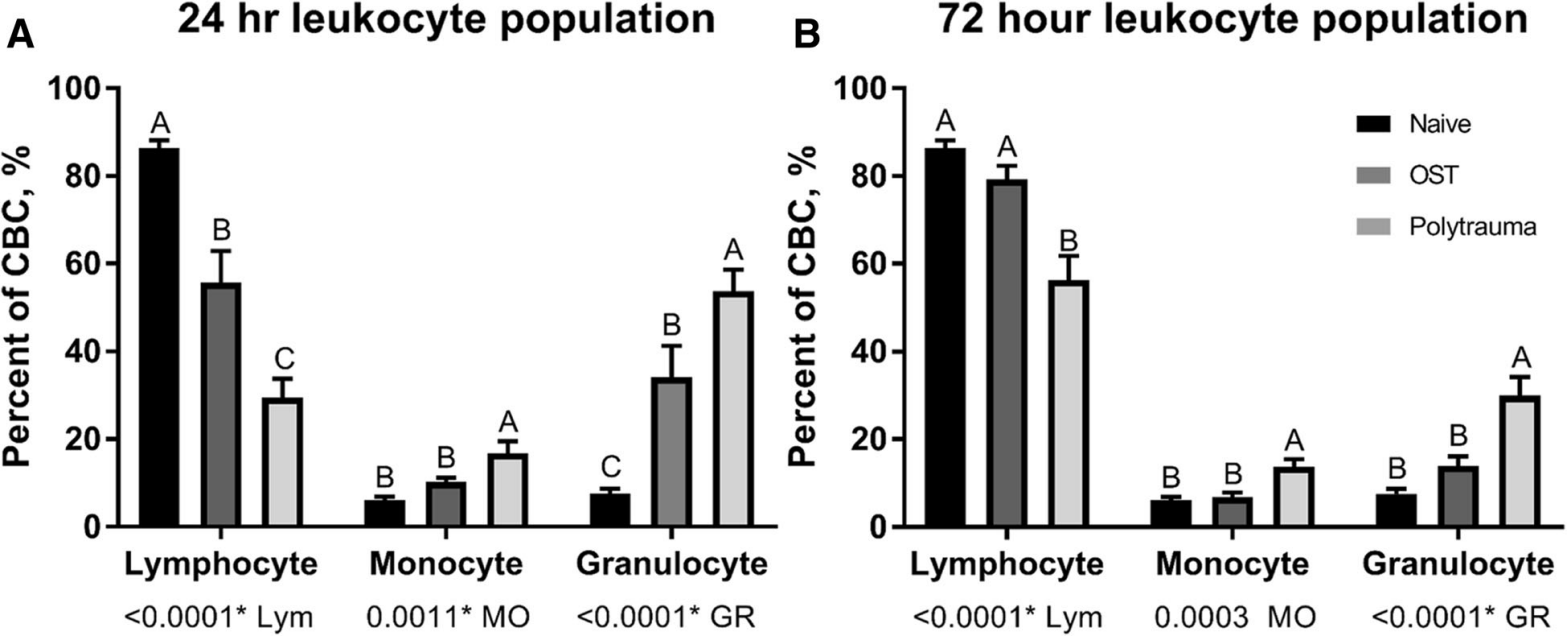
White blood cell concentration following trauma. A comparison in the percentage of total cells population identified as lymphocytes, monocytes, and granulocytes, at two time points: **a** 24 h and **b** 72 h in naïve (far left), osteotomy (middle), and polytrauma animals (far right). Values are presented as a percentage of 100 (*n* = 8/group 24 h post-injury; *n* = 7, 72 h post osteotomy; *n* = 6, 72 h post polytrauma; *n* = 8 naïve). Statistically significant difference between trauma animal cohorts within each cell type, levels not connected by the same letter are significantly different, *p* < 0.05

WBC counts were assessed in naïve animals and determined at 24 and 72 h for animals subjected to osteotomy or polytrauma. The results of this analysis are summarized in Fig. 5. WBC counts for osteotomy and polytrauma animals were significantly altered (*p* < 0.05) as compared to naïve animals. Circulating lymphocyte counts were significantly reduced in polytrauma animals both 24- and 72 h post-trauma, while monocytes and granulocytes were significantly increased compared to naïve and OST at these time points. While the total number of circulating leukocytes was reduced at 72 h in the polytrauma group compared to osteotomy (6.23 ± 1.27 vs 3.63 ± 0.22), this decrease was not significantly different (*p* = 0.071) (Fig. 6).

### Alterations in DAMPS and cytokines

**Fig. 7:**
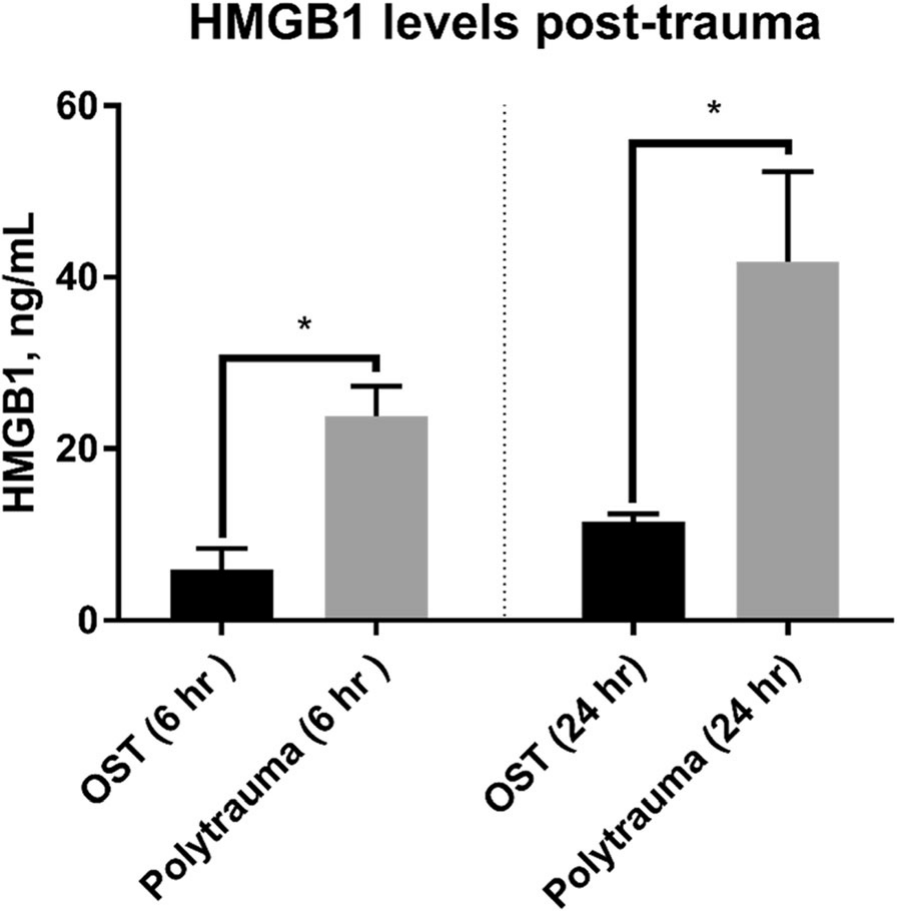
Systemic concentrations of HMGB1 at 6 and 24 h post-trauma. Plasma HMGB1 levels were measured by ELISA at 6 h (left) (*n* = 3/OST and *n* = 5/polytrauma) and 24 h post-trauma (right) (*n* = 8/OST and 5/polytrauma). At both time points, HMGB1 was significantly increased in the polytrauma group (*p* < 0.05)

HMGB1 protein was measured by ELISA (IBL International GMBH Hamburg, Germany or PeproTech, Rocky Hill, NJ) in plasma from osteotomy and polytrauma animals at 6 and 24 h post-trauma. The plasma HMGB1 levels were significantly increased (*p* < 0.05) in the polytrauma cohort when compared to the osteotomy cohort at both time points (23.82 ± 3.50 ng/ml vs 5.89 ± 2.47 ng/ml for 6 h post-injury; 41.84 ± 10.53 ng/ml vs 11.51 ± 0.91 ng/ml for 24 h post-injury) (Fig. 7).

Systemic concentrations of 23 cytokines were assessed using a multiplex immunoassay (Rat Cytokine Group 1 Panel 23 Plex, Bio-Rad Laboratories, Inc., Hercules, CA). The results of this assay are summarized in Table 1. Of note, alterations were found in many of the circulating cytokines at both the 24- and 72-h time points. Polytrauma induced significant decreases in circulating pro- and anti-inflammatory cytokines. In particular, significant reductions in human growth-regulated oncogene/keratinocyte chemoattractant (GRO/KC), interleukin (IL)-10, and macrophage inflammatory protein (MIP)-1a were observed at the 24-h time point compared to time-matched OST samples. At the same time point, significant increases were observed for IL-7, IL-12p40, and MIP-3a. Compared to OST at 72 h post-injury, polytraumatic injury induced significant decreases in circulating TNFα, IL-6, and MIP-1a. At this time, polytrauma animals exhibited a 60% increase in circulating glucagon and a 45% decrease in leptin; however, these changes failed to reach significance (*p* = 0.0556 and *p* = 0.1077, respectively).

**Table 1:** Concentration of cytokines in plasma following trauma at 24 and 72 h post-trauma

Analyte	24 h osteotomy	24 h polytrauma	*p* value	72 h osteotomy	72 h polytrauma	*p* value
G-CSF	201.00 ± 15.73	195.4 ± 14.26	0.9610	110.80 ± 22.75	67.20 ± 0.86	0.1260
Glucagon	88.76 ± 16.76	90.73 ± 7.01	0.9912	62.80 ± 3.03	105.28 ± 13.20	0.0556*
GM-CSF	53.72 ± 12.70	54.46 ± 8.679	0.9979	54.46 ± 8.68	44.53 ± 3.72	0.9777
GRO/KC	9.51 ± 1.94	3.50 ± 0.48	0.0102**	3.5 ± 0.78	1.74 ± 0.10	0.5746
IFN-γ	68.74 ± 5.82	61.61 ± 15.00	0.8757	38.38 ± 13.74	15.86 ± 4.46	0.2944
IL-1a	7.53 ± 4.28	6.39 ± 3.72	0.9597	8.33 ± 1.76	1.55 ± 0.00	0.2807
IL-1b	5.60 ± 0.47	5.69 ± 0.55	0.9896	6.24 ± 0.37	6.05 ± 0.55	0.9572
IL-2	22.28 ± 4.31	11.85 ± 1.64	0.0537*	9.40 ± 0.71	9.38 ± 2.24	0.9999
IL-4	143.28 ± 41.07	83.59 ± 34.57	0.3508	123.67 ± 18.64	45.62 ± 20.30	0.2689
IL-5	1.42 ± 0.27	2.33 ± 1.00	0.5264	2.49 ± 0.43	1.12 ± 0.19	0.2197
IL-6	104.12 ± 26.50	59.21 ± 3.63	0.1658	115.54 ± 14.72	53.27 ± 8.62	0.0330*
IL-7	59.25 ± 16.33	124.25 ± 14.36	0.0177*	ND	ND	N/A
IL-10	13.50 ± 3.15	4.86 ± 1.18	0.0240*	4.86 ± 1.06	2.14 ± 0.09	0.6749
IL-12p40	18.05 ± 2.82	38.22 ± 3.46	0.0001**	13.45 ± 0.74	10.00 ± 2.21	0.5740
IL-13	17.75 ± 1.93	10.81 ± 0.00	0.8534	26.74 ± 10.42	12.16 ± 3.77	0.1791
IL-18	11.40 ± 2.27	13.30 ± 3.04	0.7768	10.45 1.33	4.7 ± 1.07	0.1320
Leptin	604.29 ± 68.36	865.91 ± 310.39	0.5166	926.16 ± 102.66	416.43 ± 100.90	0.1077
M-CSF	2.38 ± 0.45	6.538 ± 2.645	0.7629	14.67 ± 8.054	2.47 ± .3432	0.1601
MIP-1a	5.03 ± 0.30	3.64 ± 0.30	0.0143*	5.03 ± 0.46	3.44 ± 0.12	0.0056**
MIP-2	4.25 ± 0.54	5.56 ± 1.69	0.6270	4.00 ± 1.02	2.57 ± 0.16	0.6413
MIP-3a	1.09 ± 0.14	2.00 ± 0.42	0.0405*	0.64 ± 0.22	0.64 ± 0.06	0.9995
PAI-1	13.01 ± 3.07	9.05 ± 2.13	0.3339	4.35 ± 1.00	7.29 ± 1.15	0.5350
TNF-α	69.02 ± 11.08	40.29 ± 3.67	0.7202	140.25 ± 53.69	35.19 ± 3.26	0.0313*
VEGF	65.41 ± 13.92	51.24 ± 8.67	0.6189	58.00 ± 11.73	44.91 ± 9.34	0.7323
MCP-1	17.50 ± 5.14	4.30 ± 1.00	0.1170	ND	ND	N/A

Plasma concentrations of analytes were assessed using a Bio-Plex Multiplex Immunoassay. Values are reported as picogram/milliliter in two experimental cohorts, osteotomy and polytrauma at 24 and 72 h post-trauma. Values are reported as mean ± SEM. Statistically significant differences between osteotomy and polytrauma cohorts are indicated as follows: *p < 0.05, **p < 0.01; *n* = 5/group/time point. Analytes which were non-detectable are designated as ND

### Gene expression alteration at the fracture site

mRNA expression of 84 genes related to the innate and adaptive immune response was analyzed for differential transcription between the osteotomy and polytrauma cohort. At the 24-h time point, it was found that six genes were significantly up-regulated in the polytrauma group (*p* < 0.05): C-C motif chemokine receptor 6 (*Ccr6*), C-X-C motif chemokine ligand 10 (*Cxcl10*), interferon gamma receptor 1 (*Ifngr1*), interleukin 1 receptor type 1 (*Il1r1*), NFKB inhibitor alpha (*Nfkbia*), and mitogen-activated protein kinase 8 (*Mapk8*). While the same genes were evaluated in the 72 h post-trauma group, none of the six genes that were identified at 24 h were significantly different between the osteotomy and polytrauma cohort. At the 72-h time point, the following genes were significantly downregulated in the polytrauma group: C-C motif chemokine ligand 3 (*Ccl3*), interleukin 1 beta (*Il1b*), interleukin 6 (*Il6*), Jun pronto-oncogene, AP-1 transcription factor subunit (*Jun*).

**Fig. 8:**
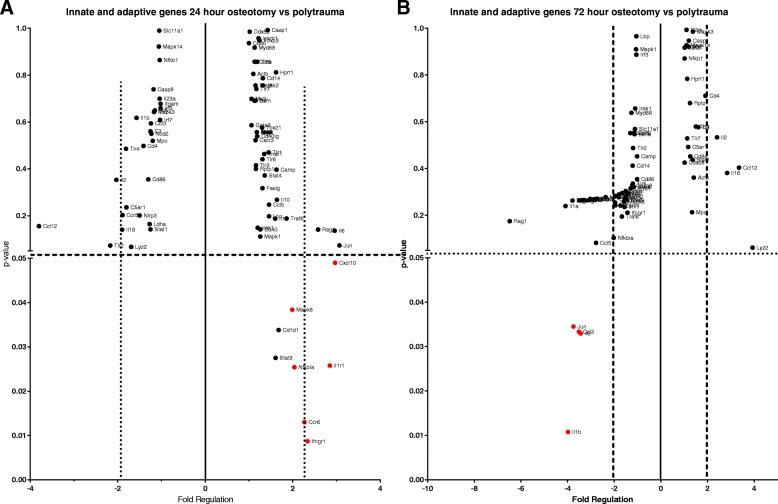
Local expression of inflammatory genes at 24 h and 72 h post-trauma. Innate and adaptive responses were compared between osteotomy and polytrauma animals in RNA isolated from the femur following trauma by RT2 PCR Array. **a** Out of 84 genes, 6 were significantly (*p* < 0.05) upregulated at least twofold in the polytrauma cohort compared to osteotomy at 24 h and **b** 4 genes were significantly downregulated at 72 h (*N* = 5/group)

Both groups were evaluated for changes to genes governing osteogenic differentiation at the 10-day time point. Out of 84 genes investigated, three genes, transforming growth factor beta receptor 3 (*Tgfbr3*), bone morphogenetic protein 6 (*Bmp6)*, and vascular cell adhesion molecule 1 (*Vcam1*), were significantly downregulated in the polytrauma group (− 1.28-fold, − 1.29-fold, and − 1.55-fold, respectively) (*p* < 0.05) (Fig. 8).

### Changes in body mass post-trauma

At 48 h post-trauma, the polytrauma cohort began to significantly decline in body mass, reported as a percent of initial surgery weight, compared to OST (93.1% versus 96.8%, respectively). While the osteotomy group never dropped below 96% of initial surgery weight and had surpassed initial body mass by week 2, the polytrauma group dropped to 89.8% of surgery weight at 72 h and did not reach initial body mass until week 5 (Additional file 1B). At 48 h and beyond, the average body mass of both cohorts were significantly different (48 h *p* < 0.01; all subsequent assessments *p* ≤ 0.0001). Despite significant decreases to the average body mass in the polytrauma cohort, daily and weekly behavior assessments of all animals did not indicate a decrease in appetite in this group.

In addition to changes in body mass, significant differences were seen in assessment scores in the categories of general appearance and behavior at the 48–56-h time points; however, both OST and polytrauma animals returned to baseline by week 2 for general appearance and 72 h for behavior (Additional file 1A).

## Discussion

Fracture healing is significantly delayed in patients with severe or multiple injuries [1, 6, 8, 21, 22]. These patients often require longer recovery times and are at risk of developing non-union; delayed wound healing is correlated with an added burden on civilian and military medical services and either delays or precludes a return to normal activity. The present study describes a polytrauma model which provides substantial levels of insult through a combination of a 3-mm segmental defect, blunt force trauma to the chest, and a full-thickness scald burn which reduced fracture healing and induced several phenotypic changes at very early time points compared to uncomplicated osteotomy including (1) reduced bone volume fraction and an early increase in a circulating cytokine mediator, (2) perturbations to local and circulating cytokine concentrations, and (3) altered patterns of cellular infiltration to the wound site and leukocyte kinetics. This model reproduces the effect of polytraumatic injuries commonly seen on the battlefield, or in severe traumatic injuries in civilian populations, as well as their impact on healing, while minimizing mortality and morbidity.

Previous animal studies examining polytrauma have demonstrated altered and delayed wound healing compared to lesser forms of trauma: impaired bone healing has been reported when the injury was accompanied by blunt chest trauma, blunt chest trauma in addition to soft tissue trauma, or when fracture was accompanied by minimal associated soft tissue damage or volumetric muscle loss [8, 21, 30, 37]. Data from the present study indicate that similar changes occur to fracture healing in our model, with bone volume fraction significantly reduced by day 35 in the polytrauma cohort. In our study, we did not achieve full union at the 5-week time point; however, this 3-mm defect is a non-critical sized defect that has been previously demonstrated to heal without intervention, though this healing may be delayed [31, 38].

Acute inflammation is an integral part of bone healing; however, unlike the acute phase inflammation that precedes healing in an uncomplicated fracture, the uncontrolled systemic immune response that has been described in polytrauma and other patient populations appears to be dysregulated and may lead to chronic, non-resolving inflammation and increased incidence of non-union [1, 4, 6]. DAMPs are responsible for initiating non-infective inflammatory responses, including cytokine secretion, and a systemic increase in HMGB1 in particular has been implicated in aberrant and immunosuppressive responses to trauma [39, 40]. Additionally, increases in inflammatory cytokines like IL-6 have been reported 24 h post-polytrauma injury, an effect that was associated with reduced fracture healing [21]. Despite early increases in systemic HMGB1 in the polytrauma group, a systemic pro-inflammatory cytokine response as a result of increased trauma reported in previous literature, particularly an increase in cytokines like IL-1, TNFα, and IL-6, was not observed in this study [41]. Instead, polytrauma animals exhibited alterations to both pro- and anti-inflammatory circulating cytokines at both the 24- and 72-h time points compared to simple osteotomy. In particular, significant decreases in potentially anti-inflammatory cytokines IL-2 and IL-10 were noted in polytrauma animals at the 24 h time point; however, this was also accompanied by both an increase in cytokines related to immune recruitment, including an increase in circulating MIP-3a and a concomitant decrease MIP-1a and GRO/KC. Further evidence for immunosuppression in the polytrauma group was apparent at 72 h, with significant reductions in circulating TNFα and IL-6. The pronounced and early increase in circulating HMGB1, followed by a decrease in pro-inflammatory cytokines at 72 h post-trauma, is in agreement with a recent study by Timmermans et al. These authors found that while plasma DAMPs were increased in multi-trauma patients prior to admission to the emergency room, circulating levels of IL-6 were decreased at 72 h post injury while TNF-α remained unchanged.

In addition to circulating cytokines, we characterized the local expression of inflammatory genes within the fracture. Interestingly, the immune response within the wound space of polytrauma animals began with a significant increase in expression levels for the receptor for macrophage inflammatory protein-3, *Ccr6* (also known as CD196), as well as the genes regulating expression of *Ilr1*, the receptor for IL1β, and *Cxcl10*, or interferon gamma-induced protein 10. These data are consistent with a study of the bone marrow of immunologically restricted patients, which found higher concentrations of IL-1β, IL-6, and IP-10/CXCL10 in the fracture hematoma of a population that later demonstrated reduced rates of bone healing [4]. By 72 h, significant decreases in local expression of *Ccl3* (also known as macrophage inflammatory protein-1), *Il6*, *Il1b*, and *Jun* were observed. The significant decrease in local expression of *Ccl3* and *Il6* match the drop in circulating cytokine levels of MIP-1a and IL-6, indicating the systemic depressed immune response is matched by a local decrease in expression of genes related to a pro-inflammatory state. In general, the temporal patterns of cytokine expression indicated a depressed immune response that did not favor either a systemic pro- or anti-inflammatory response. The severity of the injuries seen in this model may indicate a state of immune hyporesponsiveness that has been previously described in ICU patients [42–44].

Other groups have reported changes to histological analysis was performed to quantify immune cells within the fracture and at the pin sites connecting the polyacetyl plate to the femur. In our study, macrophages were not observed within the fracture gap at 24 or 72 h post-injury, while neutrophils were significantly reduced in the polytrauma within the fracture gap at 24 h. Due to the removal of the periosteum at the time of surgery, periosteal thickness could not be quantified at the fracture gap or pin sites. Formation of granulation tissue at the pin sites was used to assess the mitotic rate; however, there were no significant differences in the formation of granulation tissue between the groups. Furthermore, we observed reduced macrophage numbers at the pin sites at the 24-h time point. In our study, histological evaluation of macrophages and osteoclasts in the fracture gap and the pin sites tended to be reduced in the polytrauma group, though this finding was only significant for macrophages at the pin site at 24- h, and neutrophils were only reduced at the pin site at 24 h. This delay in macrophage migration into the wound site may contribute to delayed fracture healing, as others have found that macrophage depletion during the early anabolic phase of fracture healing resulted in reduced callus formation, a process that could be completely abolished when macrophages were depleted at the time of fracture [45]. We sought to compare our results to a previous study by Recknagel et al. (2013), which described disparate immune cell infiltration patterns into the fracture callus, as well as an impaired bone fracture healing in a rat model of blunt chest trauma and osteotomy when compared to an isolated fracture [8]. Specifically, these authors found alterations in PMN and monocyte/macrophage infiltration into the periosteal callus, but not the fracture gap, in osteotomy and blunt chest trauma models. Changes to PMN infiltration were noted 3 days post-fracture, while monocyte/macrophage cell recruitment was impaired until at least day 7 in the polytrauma animals. Additionally, Recknagel et. al. (2013) found no alterations to the number of osteoclasts in the polytrauma group, whereas our results indicated a significant decrease in osteoclasts within the fracture gap. The authors of this study concluded that a polytraumatic injury alters the recruitment of inflammatory cells and cytokines at the site of the fracture, impairing wound healing [8]. While our study time points do not exactly align with the aforementioned study, the present results indicate perturbed temporal patterns of cellular infiltration that may contribute to delayed healing.

Previous work by our laboratory reported delayed fracture healing was shown by the additive effects of trauma in a less severe model of injury involving volumetric muscle loss and tibial fracture [33]. Studies of fracture hematomas from immunologically restricted patients exhibiting reduced fracture healing have also found altered immune cell populations within both the fracture hematoma and the surrounding bone marrow [4]. It has been hypothesized that neutrophilia, in combination with neutrophil priming, can induce an increase in the number of neutrophils infiltrating into the fracture hematoma, delaying downstream healing [1]. While we did not observe alterations in the neutrophil numbers within the fracture gap, we did observe a delay in the migration of macrophages and neutrophils to the pin sites near the fracture itself as well as an increase in the number of circulating granulocytes, a finding that may implicate altered local immune cell kinetics and is a possible contributing factor to delayed healing. It is important to note differences from previous reports in immune cell enumeration within the fracture callus could be due to divergent methodologies, as other groups were unable to detect differences within the fracture gap and instead quantified changes to immune cell populations within the periosteal callus [8]. Immune cells and subsequent cytokine signaling within the fracture hematoma or the periosteum are important for initiating fracture repair, as evidenced by the delay or loss of bone union when the fracture hematoma or periosteum is removed [46, 47]. The methodology described in our segmental defect model involves the stripping of the periosteum in order to accurately assess healing outcomes without periosteal interaction. This procedure mimics commonly used surgical approaches performed by clinicians when internal fixation is utilized for stabilization. It is our belief that performing internal stabilization will provide a model that is better suited for translational comparison to improve clinical guidelines. Additionally, internal stabilization and external fixation provides different fixation stiffness which has been shown to heal through different mechanisms. Grundnes and Reikerås demonstrated that loss of the fracture hematoma [46] 2–4 days after injury can reduce bone rigidity, while Ozaki et al. and Utvag et al. reported that loss of the periosteum or bone marrow [47, 48] can delay bone union. However, the authors found that even loss of bone marrow or periosteum did not abolish healing, as histological evaluation demonstrated that these rats still exhibited regeneration similar to untreated rats by day 6, indicating that our trauma model may cause systemic responses that tip the balance of healing in a manner that does not facilitate recovery.

Previous work involving polytrauma patients have found alterations in the circulating leukocyte numbers for 2 weeks following injury [6]. In our current study, we observed perturbations in the peripheral white blood cell counts that were dependent upon the level of trauma sustained, as detailed in Fig. 6. In human patients, significant alterations in peripheral blood leukocyte kinetics have also been noted in multi-trauma patients with impaired and normal tibial fracture healing outcomes [6]. The investigators noted a significant reduction in the total leukocyte counts that continued over a 2-week period in patients that exhibited impaired fracture healing, which is in agreement with our findings in the polytrauma groups. Furthermore, it has been hypothesized that bone marrow failure in trauma patients may contribute to the loss of WBC function and the perturbations often seen in the immune responses of injured patients and animals [49]. While our present study did not evaluate the circulating leukocytes beyond 72 h, we did see a reduction in the total number of WBCs at this time point, possibly indicating a similar trend in leukocyte kinetics.

Lastly, previous studies found that blunt chest trauma did not induce significant weight loss [8, 21]; however, our polytrauma model did exhibit significant weight loss following trauma, as well as impaired weight gain over the course of 35 days, with no observable difference in appetite between the groups, indicating that the additional trauma of burn may induce a more severe injury characterized by a catabolic state often seen in polytrauma patients [50]. In particular, a 15–20% total body surface area burn is associated with impaired immune response and initiates a catabolic state [51, 52]. The dysregulated immune response, as seen in the present study and in burn patients, is often accompanied by a hypermetabolic state in which energy use, insulin resistance, and oxygen demand are all increased [50]. In this hypermetabolic state, energy is used to lyse skeletal muscle to assist with gluconeogenesis in order to combat insulin resistance, instead of being used for fracture healing [50]. The alterations in glucagon and leptin seen at 72 h post-trauma indicate possible early stage metabolic perturbations.

## Conclusions

Altogether, our study indicates that the polytrauma model described herein is one of altered systemic and local immune responses, but not necessarily one of systemic inflammation. The local immune and systemic responses in our polytrauma model differ from that of osteotomy alone in profound ways and may be one of the primary factors for the reduced bone volume fraction seen in the polytrauma group. Further investigation is required to better understand the immune responses seen in our study, while testing of intervention strategies, which are severely lacking for polytrauma patients, may help inform clinical guidelines to improve return to duty in severely injured service members along with civilian trauma. We feel that the model described herein represents a valid surrogate of human polytrauma and multi-trauma that will be useful to future research and development of interventional strategies focusing on methods to improve wound healing through immunomodulation.

## Additional file

Additional file 1: Assessment Scores. Description: A) Average assessment scores for general appearance over the 5wk survival period B) Average percent weight loss over the 5wk survival period C) Average assessment scores for behavior over the 5wk survival period D) Assessment score rubric. (PPTX 135 kb)

## Abbreviations

Bmp6: Bone morphogenetic protein 6
Ccl3: C-C motif chemokine ligand 3
Ccr6: C-C motif chemokine receptor 6
Cxcl10: C-X-C motif chemokine ligand 10
DAMPs: Damage-associated molecular patterns
ELISA: Enzyme-linked immunosorbent assay
GRO/KC: Human growth-regulated oncogene (GRO)/keratinocyte chemoattractant (KC)
H&E: Hematoxylin and eosin
HMGB1: High mobility group box 1 protein
Ifngr1: Interferon gamma receptor 1
IL: Interleukin
Il1b: Interleukin 1 beta
Il1r1: Interleukin 1 receptor type 1
IR: Immunologically restricted
Jun: AP-1 transcription factor subunit
K-wire: Kirschner wire
Mapk8: Mitogen-activated protein kinase 8
MIP-3a: Macrophage inflammatory protein
mRNA: Messenger ribosomal nucleic acid
Nfkbia: Nuclear factor kappa-light-chain-enhancer of activated B cells inhibitor alpha
OST: Osteotomy
PCR: Polymerase chain reaction
PMN: Polymorphonuclear neutrophils
RNA: Ribosomal nucleic acid
ROI: Region of interest
RT: Room temperature
TBI: Traumatic brain injury
Tgfbr3: Transforming growth factor beta receptor 3
TNF-α: Tumor necrosis factor-α
Vcam1: Vascular cell adhesion molecule 1
WBC: White blood cell
μCT: Microcomputed tomography
