# Supplementary material for: Burn and thoracic trauma alters fracture healing, systemic inflammation, and leukocyte kinetics in a rat model of polytrauma
Source: J Orthop Surg Res. 2019 Feb 19;14:58. doi: 10.1186/s13018-019-1082-4 (PMC6381742; doi:10.1186/s13018-019-1082-4)
Supplement: Supplementary file 1 — Assessment Scores. Description: A) Average assessment scores for general appearance over the 5wk survival period B) Average percent weight loss over the 5wk survival period C) Average assessment scores for behavior over the 5wk survival period D) Assessment score rubric. (PPTX 135 kb) [file 13018_2019_1082_MOESM1_ESM.pptx]

## Slide 1
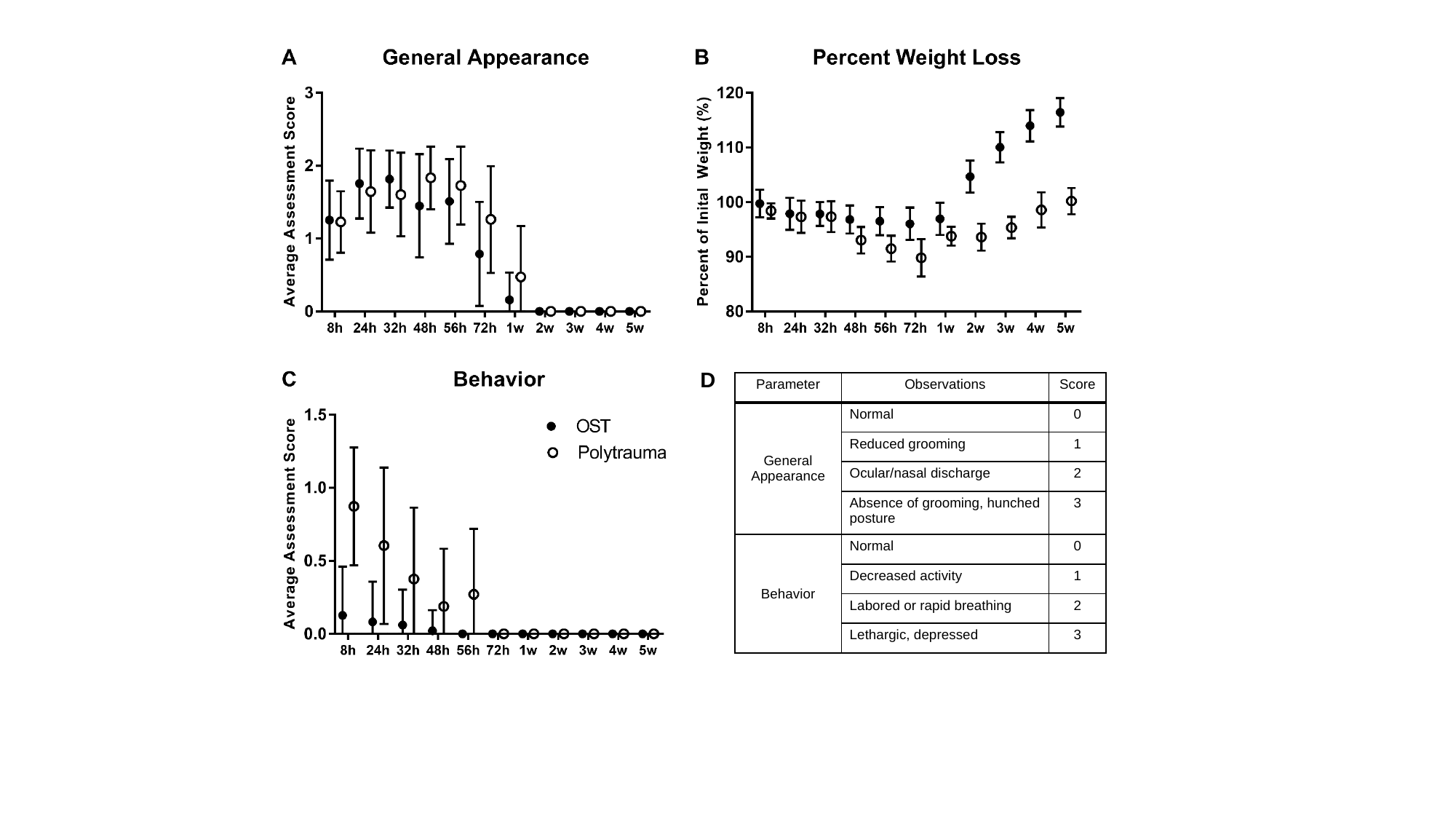

D
| Parameter | Observations | Score |
| --- | --- | --- |
| General Appearance | Normal | 0 |
| | Reduced grooming | 1 |
| | Ocular/nasal discharge | 2 |
| | Absence of grooming, hunched posture | 3 |
| Behavior | Normal | 0 |
| | Decreased activity | 1 |
| | Labored or rapid breathing | 2 |
| | Lethargic, depressed | 3 |
